# Machine learning reveals ferroptosis and therapeutic targets in rheumatoid arthritis

**DOI:** 10.1097/MD.0000000000044384

**Published:** 2025-09-26

**Authors:** Ping Xia, Hang Du, Yue Ren, Qu Zhang, Ying Shi

**Affiliations:** a Traditional Chinese Medicine Department, The Affiliated Dazu’s Hospital of Chongqing Medical University, Chongqing, China; b Department of Pharmacy, The Affiliated Dazu’s Hospital of Chongqing Medical University, Chongqing, China; c Department of Rheumatology, The Ninth People’s Hospital of Chongqing, Chongqing, China .

**Keywords:** bioinformatic analysis, ferroptosis, kaempferol, rheumatoid arthritis, Taohong Siwu Decoction

## Abstract

A complex, multisystem, chronic autoimmune disease, rheumatoid arthritis (RA) severely impairs a patient’s quality of life and finances. Though the precise chemical process is unclear, ferroptosis, a recently studied method of cell death, plays a significant role in RA. Furthermore, despite the fact that Chinese medicine, particularly the usage of Taohong Siwu Decoction (THSWD), has substantial benefits in treating RA, there are numerous shortcomings in the way RA is currently treated. In order to determine the primary acting components of THSWD and the primary acting targets that influence RA Ferroptosis based on network pharmacology, we will use machine learning to identify the major targets and molecular processes of Ferroptosis in RA. The main components and underlying molecular mechanisms of THSWD treatment for RA were elucidated by molecular docking techniques and in vitro experiments. Ferroptosis marker genes were found to be PTGS2, SLC40A1, TF, TFRC, FTH1, GPX4, HSPB1, and NFE2L2 in RA in microarray data analysis. Furthermore, we used ATF3, EGR1, and KDM6B as the ferroptosis activator genes. Ferroptosis inhibitor genes included ZFP36, NR4A1, CDKN1A, ABRD4, and RRM2. The disease process of RA may be linked to intracellular iron-ion homeostasis, iron-ion transport, and oxidative stress response, according to bioinformatic study. KEGG bioprocesses indicate that ferroptosis, the HIF-1 signaling pathway, and the p53 signaling pathway are closely linked to the development of RA. Lastly, using network pharmacology and vitro experiment, we discovered that kaempferol, a crucial part of THSWD, controlled ferroptosis to enhance RA through TFRC, ZFP36, PTGS, CDKN1A, and ATF3. The multi-component, multi-target, and multi-pathway features of THSWD for RA were demonstrated by this research investigation, which further elucidated the mechanism of THSWD for RA treatment with fresh perspectives.

## 1. Introduction

An intricate, long-lasting, multisystem autoimmune condition, rheumatoid arthritis (RA) mostly affects flexible joints.^[1]^ Out of the 100,000 new instances of RA that are reported each year worldwide, 0.5% to 1% of people are affected.^[2,3]^ Although the exact cause of RA is still unknown, the damaging joint characteristics and persistent, progressive inflammation are sometimes referred to as the invasive inflammatory tissue perpetuation of vascular opacification tissue.^[4]^ Furthermore, the development and course of RA are influenced by a number of inflammatory cells found in the synovium, such as monocytes/macrophages, T and B cells, osteoclasts, dendritic cells, endothelial cells, and synovial fibroblasts.^[5]^ While the symptoms of osteoarthritis and RA-like disease are similar, the pathophysiology of the synovium is distinct, osteoarthritic synovium has less leukocyte infiltration, while RA is characterized by synovial cell hypertrophy and hyperplasia with lymphocytic and inflammatory cell infiltration.^[6]^ Even with great progress in our knowledge of the pathophysiology of RA, early diagnosis, treatment, and underlying etiology are still difficult to achieve.

Ferroptosis is a novel iron-dependent mechanism of regulating cell death that was first proposed in 2012. It differs from traditional forms of cell death in its morphology, mechanism, induction, and inhibition. The primary mechanism is the degradation of lipid peroxides by glutathione (GSH) dependent glutathione peroxidase (GPX4), which can result in the accumulation of iron-ion-mediated lipid reactive oxygen species (ROS) when GPX4 fails. Ferroptosis is caused by the buildup of free radicals.^[7]^ Numerous signaling pathways have been discovered to control ferroptosis in a range of diseases as research on the condition continues to progress. More investigation into the Ferroptosis pathway will enhance our understanding of the mechanisms underlying RA and offer fresh concepts and potential therapeutic targets for the prevention and treatment of the condition.^[7–9]^

Taohong Siwu Decoction (THSWD), derived from Dehua Chai “*Gynecological Bingjian*” during the Qing Dynasty, is referred to as “nourishing the blood, invigorating the blood and removing blood stasis” and is made up of Radix Rehmanniae Praeparata (*Sheng Dihuang*), Radix Paeoniae Alba (*Bai Sao*), Rhizoma Ligustici (*Chuanxiong*), Rhizoma Polygonatum (*Tao Ren*), Radix Angelicae Sinensis (*Dang Gui*), and Rhizoma Polygonatum (*Hong Hua*). THSWD, which has emerged as one of the most popular formulas for reviving blood circulation and eliminating blood stasis in Chinese medicine clinics, can be used to treat RA and osteoarthritis, which are categorized in Chinese medicine as bone paralysis. It is also a common formula for treating multiple diseases at once.^[10]^ The synergistic effect of TCM’s numerous components, targets, and pathways accounts for its therapeutic success, despite the compound’s complicated chemical makeup. With the primary goal of nourishing the blood and promoting circulation to eradicate blood stasis, THSWD has been used to treat RA. The main questions to be investigated in the contemporary use of this formula are the interactions between the components, targets, pathways, and diseases.^[11]^

The molecular of RA has been the subject of numerous research approaches recently. Among these research methods, high-throughput microarray techniques have drawn a lot of interest and advanced significantly in fields like medical oncology. They have important clinical applications ranging from patient stratification to prognosis prediction, from molecular diagnosis to molecular classification, and from finding new drug targets to response prediction.^[12,13]^ The aim of this study was to analyze RA gene proteins and related BPs using a bioinformatics, network pharmacology approach to elucidate potential mechanisms for the treatment of RA with THSWD.

## 2. Materials and methods

### 2.1. Differential gene screen

The basic open-source sequencing database, gene expression omnibus (https://www.ncbi.nlm.nih.gov/geo/), compiles a vast amount of microarray and second-generation sequencing data that research institutes worldwide submit for free use by researchers anywhere. Searching for the study target’s keywords will yield relevant datasets.^[14]^ After screening, the microarray data of RA GSE55457 was obtained by searching for “RA” in gene expression omnibus.^[15]^ The data was released on March 5,2014. The approximate time range of the analysis in this article is: December 2024. The online bioinformatics analysis tool Sangerbox 3.0 was used for the subsequent analyses. For the analysis, the R package umap (version 0.2.7.0) was utilized. First, the z-score of the GSE55457 expression profile was determined. Then, the reduced matrix was obtained by doing additional dimensionality reduction using the umap function.

The “limma” tool was chosen for differential gene analysis following initial data availability verification. The data were first log2 transformed, and multiple linear regression was carried out using the lmFit function. Additionally, the eBays function was utilized to compute the moderated t-statistics, moderated F-statistic, and log-statistic. Calculate the log-odds of differential expression, the moderated t-statistic, and the moderated F-statistic using the empirical Bayes moderation of the standard errors toward a common value. Finally, determine the differential significance of each gene.

### 2.2. Weighted network construction

A systems biology technique called weighted gene co-expression network analysis (WGCNA) is used to describe patterns of gene association between various samples. This can be used to find gene sets that differ greatly in synergy, as well as to find potential biomarker genes or therapeutic targets based on gene-set introgression and gene-set-phenotype associations.^[16,17]^ Here is the precise procedure: use weighted expression correlation to build a gene co-expression network. Determine gene sets by performing hierarchical clustering analysis based on the weighted correlation and slicing the clustering results based on the predetermined criteria to produce distinct gene modules, which are represented by the clustering tree’s branches and various colors. Determine trait-related modules and, if phenotypic data is available, compute the correlation between gene modules and phenotypes. Examine how models relate to one another and examine the system-level interoperability network of various models. Choose driver genes of interest from important models, or make assumptions about the role of unknown genes by looking at the roles of known genes in the models.

### 2.3. Ferroptosis gene screening

For the management and characterization of ferroptosis-related indicators and regulators as well as ferroptosis-associated disorders, FerrDb (http://www.zhounan.org/ferrdb/current/) is currently the first manually managed ferroptosis database.^[18]^ The FerrDb database provided the marker gene, activator gene, and repressor gene data used in this investigation. To identify the flag targets of a possible Ferroptosis mechanism in RA, the illness data of RA were first intersected with the Ferroptosis marker genes. Second, RA-activated targets with clinical predictive significance were screened by combining Ferroptosis-activated genes with modular genes and differential genes. Similar to this, RA inhibitory targets with clinical predictive relevance were found by screening for the intersection of Ferroptosis inhibitory genes with differential genes and modular genes. The tagged, activated, and inhibited targets of RA were then used to build a frontal interaction network amongst targets. After screening for Ferroptosis-associated genes in RA, we again returned the genes to the microarray data, extracted the raw data, constructed and validated the differential expression of the relevant genes in RA, and the final results are presented in a differential box-and-line plot.

### 2.4. Bioprocess analysis

We obtained the most recent gene annotations of the KEGG Pathway as background for the gene-set functional enrichment analysis using the KEGG rest API (https://www.kegg.jp/kegg/rest/keggapi.html). We then mapped the genes to the background set and carried out the enrichment analysis using the R software package clusterProfiler (version3.14.3) to obtain gene-set enrichment results.^[19,20]^ A *P*-value of <0.05 and an FDR of <0.25 were deemed statistically significant, and the minimum gene set was set at 5, and the maximum at 5 thousand. The 3 components of gene function are cellular component (CC), molecular function (MF), and biological process (BP), according to GeneOntology. We can determine the primary relationships between our target genes at the CC, MF, and BP levels by using the GO database. Likewise, to acquire the gene-set enrichment results, enrichment analysis was carried out using the R package clusterProfiler (version 3.14.3).^[21]^

### 2.5. Immune infiltration analysis

RNA-Seq and microarray data can be supported by ImmuneCellAI, a tool for predicting the number of various immune cells from gene expression data. It can forecast cancer immunotherapy responses, assess data from humans and mice, and deal with population-specific immune cell variations.^[19]^ The R package, which involves consideration of data pretreatment and grouping information settings, allows users to study the data. ImmuneCellAI can forecast a patient’s reaction to immune checkpoint inhibitor medication and estimate the proportions of 18 T-cells and 6 additional immune cell types. The present study was analyzed using this immunoassay tool.

### 2.6. THSWD active compound screening

The first database to systematically collect blood constituents of TCM prescriptions and herbs is DCABM-TCM, a database of substances absorbed into blood and metabolites of traditional Chinese medicine.^[20]^ At DCABM-TCM, we gathered THSWD drug enrollment components and screened the same compounds from several medications as key compounds. An electronic chemical database for spectral/structural deposition, retrieval, search, and analysis is available online at NP-MRD. Online assignments and spectral/structural visualization tools help make the data easy to understand. To confirm the medications’ chemical makeup, we constructed mass spectral peak plots of the selected main components.

### 2.7. Mechanism of THSWD for rheumatoid arthritis excavation

The origin and source of important medications were clarified in the TCM database, and the chemical structures of important compounds were built based on the screening results of THSWD active substances. The target of action and primary mediators of action of important chemicals can be predicted using the Swisstargetprediction (http://www.swisstargetprediction.ch/?) program.^[22]^ The hub gene of THSWD for RA was constructed based on the intersection of the Ferroptosis gene and the action targets of joint medications. The hub gene was then examined using KEGG enrichment once more to elucidate the signaling pathway of THSWD for RA.

### 2.8. Validation by in vitro cell experiments

#### 2.8.1. Preparation of kaempferol solution

Dissolve 10 mg of kaempferol in 438 μL DMSO into 100 mmol/L stock solution, split and stored at −20℃. Complete medium was diluted to 0.01,0.1,1,10, and 100 μmol/ L before treatment.

#### 2.8.2. Cell culture system

Human synovial fibroblasts were cultured in a 37℃ constant-temperature incubator containing 5% CO₂, and all experiments were performed at cell confluence above 80%. Medium was prepared by complete medium with commercial human synovial fibroblasts.

#### 2.8.3. Detection of cell proliferation (CCK-8 method)

##### Cell treatment

After cells reached 80% confluence, single cell suspension was prepared with digestion solution containing 0.25% trypsin, seeded to 96-well plates at a density of 3000 cells/ well, and pre-cultured at 37℃ for 24 hours.

##### Drug intervention

Change the medium containing different concentrations of kaempferol (0.01–100 μmol/L) with 200 μ L per well for 24 and 48 hours.

##### Assay

10 μL CCK-8 reagent was added to each well, and after further incubation for 2 hours, the absorptive value at 450 nm was measured by a microplate reader.

#### 2.8.4. Verification by qRT-PCR

##### Experimental group

Set up normal control group, RA model group and RA treatment group (chondrocytes intervened with 10 ng/mL IL-1 β to create RA cell model and construct the model group.) Antibodies for ATF3, ZFP36, PTGS2, TFRC and CDKN1A were purchased from Baiji Corporation (Shanghai, China). The supplier of CCK-8 kit is Beijing Solaibao Technology Co., Ltd.

##### RNA extraction

Total RNA was isolated from the 3 cell groups using a total RNA extraction kit.

##### Reverse transcription

cDNA templates were prepared using the iScript cDNA synthesis kit.

##### Quantitative analysis

qPCR was performed using the Bio-Rad CFX96 system with GAPDH as the reference gene, and the relative mRNA expression was calculated using the 2 ^ - ΔΔ Ct method.

## 3. Results

### 3.1. differential gene identification

**Figure 1.**
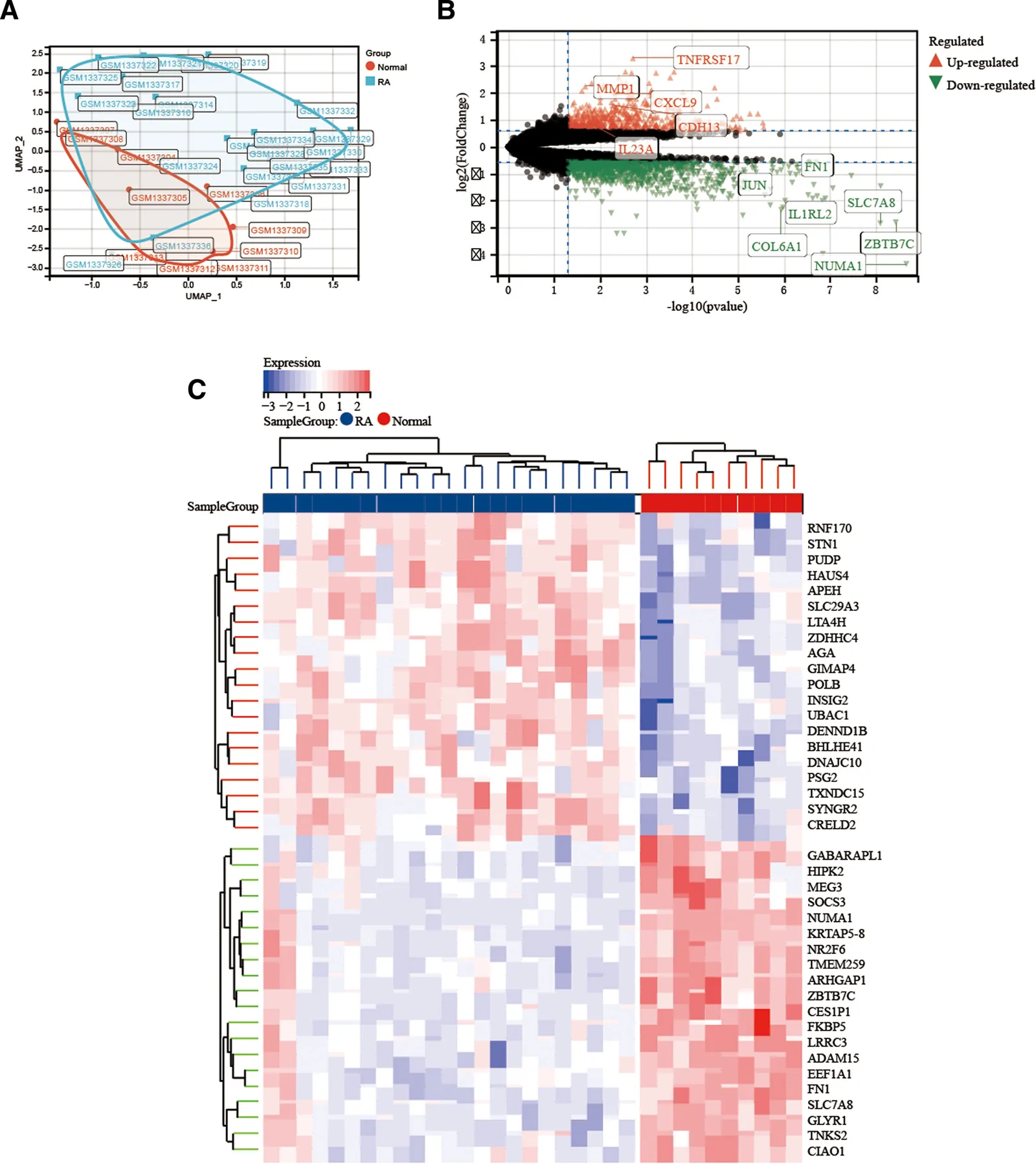
(A) Downscaling analysis of microarray data, (B) Differential volcano map genes of GSE55457, (C) Differential heat map genes of GSE55457.

It was first confirmed that the RA group and the normal group were dispersed in distinct 2-dimensional spaces and that the data were distinct from one another using the dimensionality reduction analysis of the microarray data GSE55457, as illustrated in Figure 1A. Genes with substantial differences, including 604 up-regulated genes and 975 down-regulated genes (Fig. 1B) (*P < .05, FDR < 0.05), were ultimately acquired by comparing the difference volcano plots. Table 1 displays the remaining differential gene data with various thresholds. The expression of the top 20 genes in various samples was further demonstrated by the differential gene heatmaps. Lastly, we discovered that JUN, FN1, and SLC7A8 were considerably down-regulated genes, whereas MMP1, IL23A, and CXCL9 were significantly up-regulated genes based on the heatmap and volcano plot analysis (Fig. 1C, Table S1 and 2, Supplemental Digital Content, https://links.lww.com/MD/Q55).

**Table 1:** Differential genes with different thresholds.

Significance threshold	Comparable group	Control group	1.2 Times		1.3 Times		1.5 Times		2 Times	
			Up	Down	Up	Down	Up	Down	Up	Down
*P* < .05	RA	Normal	1448	1823	1087	1530	604	975	208	312
*P* < .01			746	1022	628	938	364	667	151	256
FDR <0.05			573	811	500	758	300	557	124	223
FDR <0.01			133	280	131	276	87	217	29	112

### 3.2. Construction of weighted gene network

**Figure 2.**
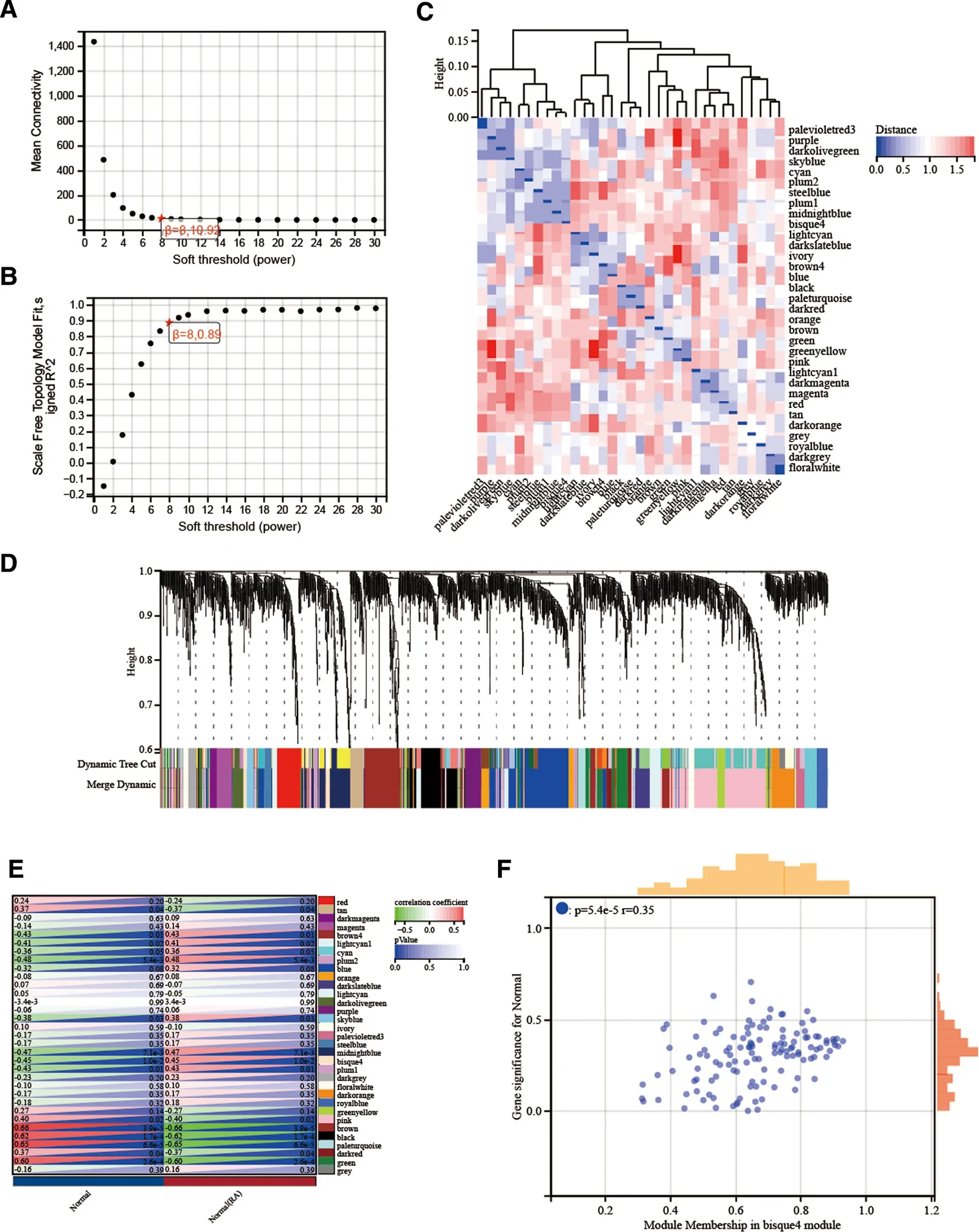
(A) When the parameter β of the scale-free network is 8, the average connection value is 10.92, (B) When the parameter β of the scale-free network is 8, the independence reaches 0.89, (C) The correlation heatmap between modules demonstrates the connection between different modules, (D) When the cut height is set to 0.25, the gene clustering tree shows that a total of 33 functional modules were clustered into the (E) The bisque4 module is positively correlated with the RA correlation (correlation coefficient = 0.45, *P* = 1.0e-2), (F) The scatter plot of GS and MM correlation also suggested that the bisque4 module was positively correlated with RA (*P* = 5.4e-5, *R* = 0.35).

**Figure 3.**
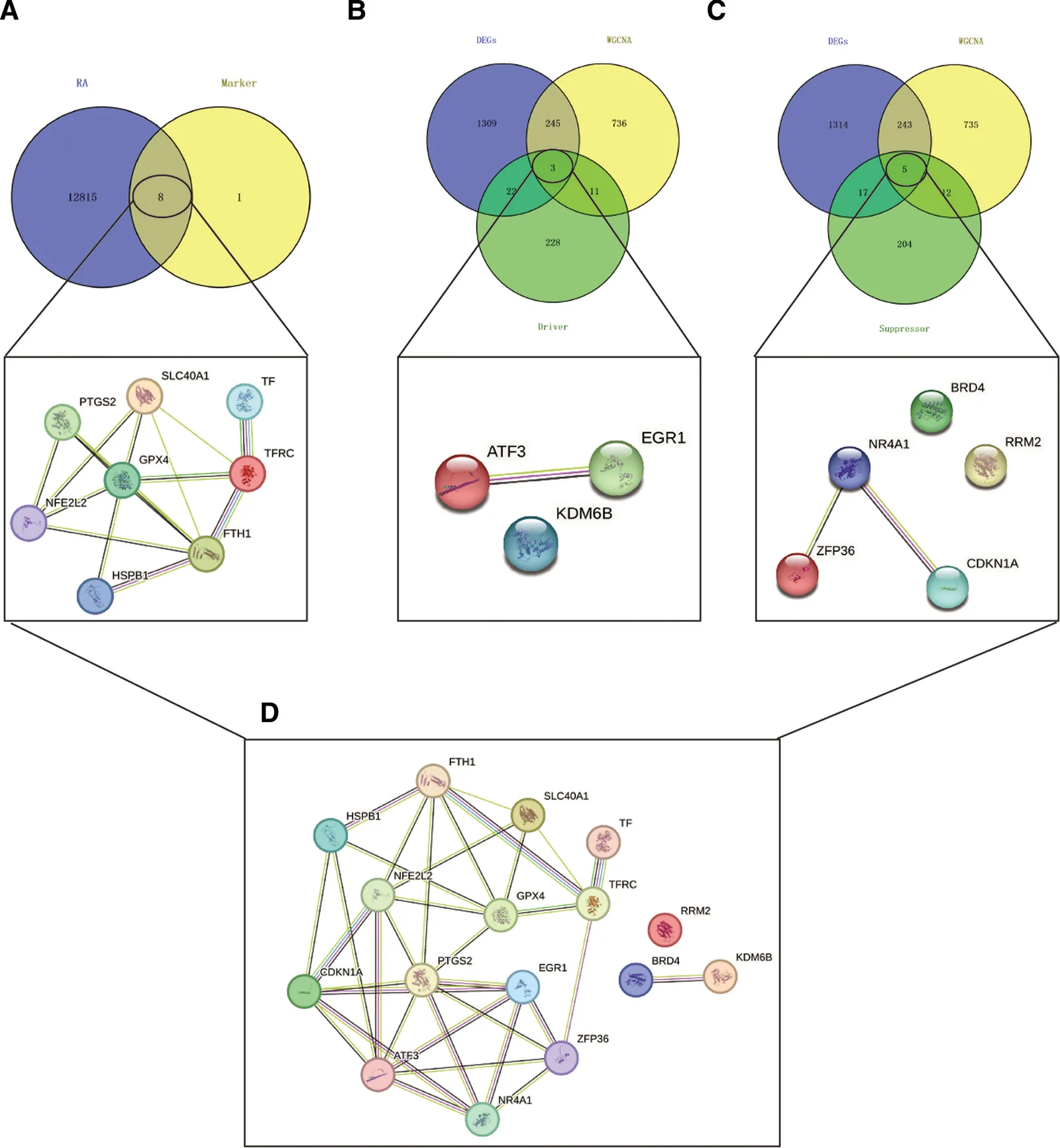
(A) Intersection of iron death marker genes with microarray data in rheumatoid arthritis, (B) Intersection of iron death activation genes with differential and modular genes, (C) Intersection of iron death repressor genes with differential and modular genes, and (D) Network diagram of iron death marker genes, repressor and activator genes in rheumatoid arthritis.

The microarray data GSE55457 was used to create a weighted gene co-expression network after the aberrant samples were eliminated. The data demonstrated a solid linear association between the genes when the scale-free network’s parameter β was set to 8. The average linkage value was 10.92 and the independence reached 0.89 (Fig. 2A and B). The gene clustering tree revealed that 33 functional modules were clustered overall when the cut height was set to 0.25 (Fig. 3D). The correlation heatmap between modules illustrated the relationships between the various modules (Fig. 3C), and the modules were significantly associated. The correlation and confidence of various modules with diseases were further illustrated by the contrast heatmaps of modules with various sample groups. Figure 2E shows a favorable association between the bisque4 module and RA (correlation coefficient = 0.45, *P* = 1.0 × 10^−2^). The bisque4 module appeared to have a favorable connection with RA (*P* = 5.4 × 10^−5^, *R* = 0.35), according to the GS versus MM correlation scatter plot (Fig. 2F). Lastly, 995 module-associated genes were identified (Table S3, Supplemental Digital Content, https://links.lww.com/MD/Q55).

### 3.3. Screening of key genes for RA ferroptosis

**Figure 4.**
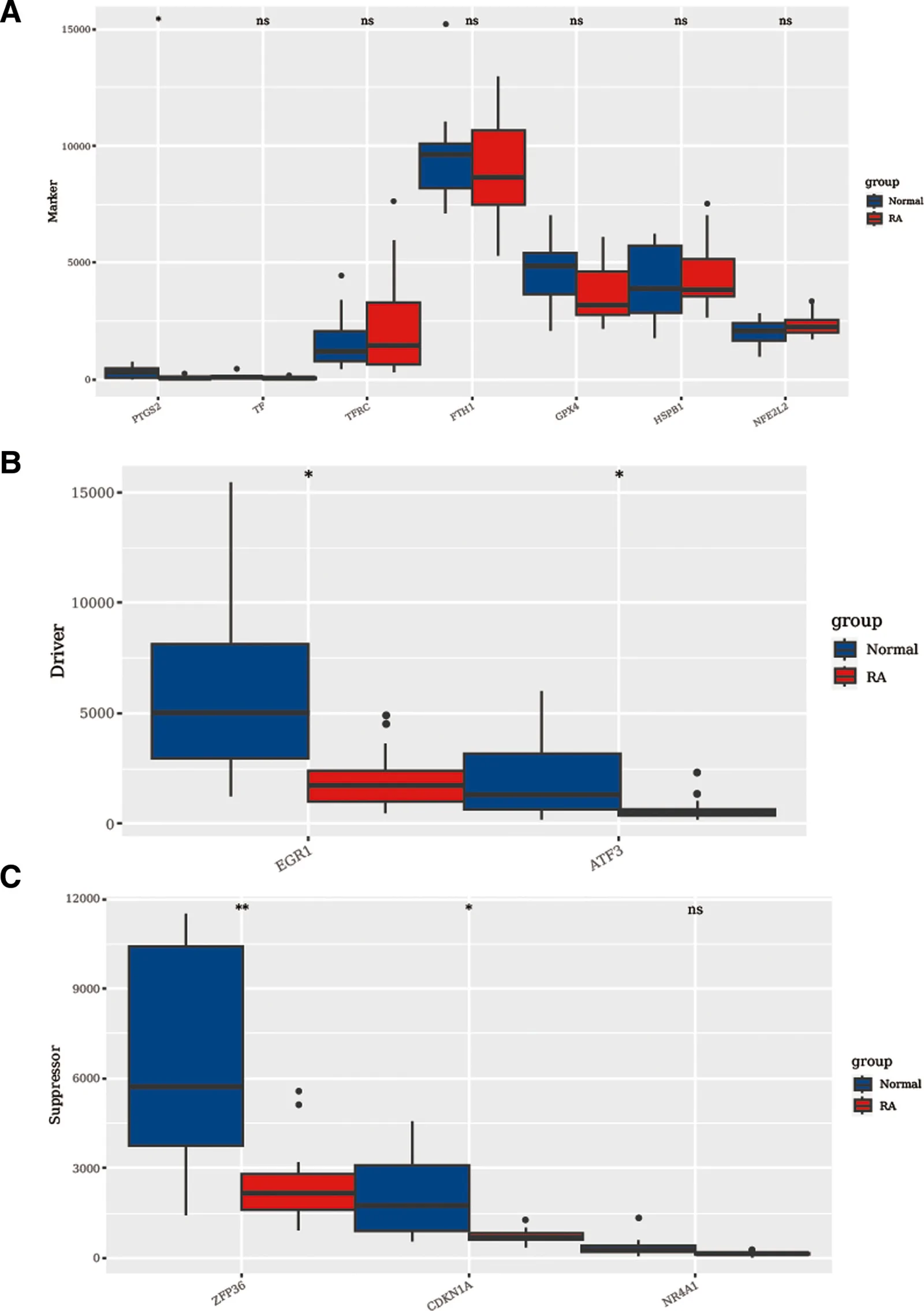
(A) Statistical analysis of iron death marker genes in rheumatoid arthritis, (B) Statistical analysis of iron death activation genes in rheumatoid arthritis, and (C) Statistical analysis of iron death suppression genes in rheumatoid arthritis.

We obtained 8 Ferroptosis marker genes for RA by downloading the essential genes for Ferroptosis and analyzing the microarray data for both RA and Ferroptosis marker genes. These were PTGS2, SLC40A1, TF, TFRC, FTH1, GPX4, HSPB1, and NFE2L2 (Figure 3A, Table S4, Supplemental Digital Content, https://links.lww.com/MD/Q55). Although these results were not statistically significant, additional statistical analysis revealed that the sample tissues had high expression levels of TFRC, FTH1, GPX4, HSPB1, and NFE2L2 (Fig. 4A). In contrast, FTH1 and GPX4 displayed a declining trend in RA, whereas TFRC, HSPB1, and NFE2L2 displayed a rising trend. This is in line with previous studies that indicate a decline in GPX4, a key enzyme in the control of the glutathione system, an antioxidant system, at the initiation of ferroptosis. ATF3, EGR1, and KDM6B were the 3 Ferroptosis activators that we also examined. The tissue expression of ATF3 and EGR1 in the sample was statistically significant (Figs. 3B and 4B). Ferroptosis was inhibited by ZFP36, NR4A1, CDKN1A, and ABRD4, RRM2, of which sample tissues expressed ZFP36, CDKN1A, and NR4A1 (Figs. 3C and 4C). Finally, based on Ferroptosis-associated genes, we created an association network of these genes using the STRING database (Fig. 3D).

### 3.4. Bioinformatic analysis of RA

**Figure 5.**
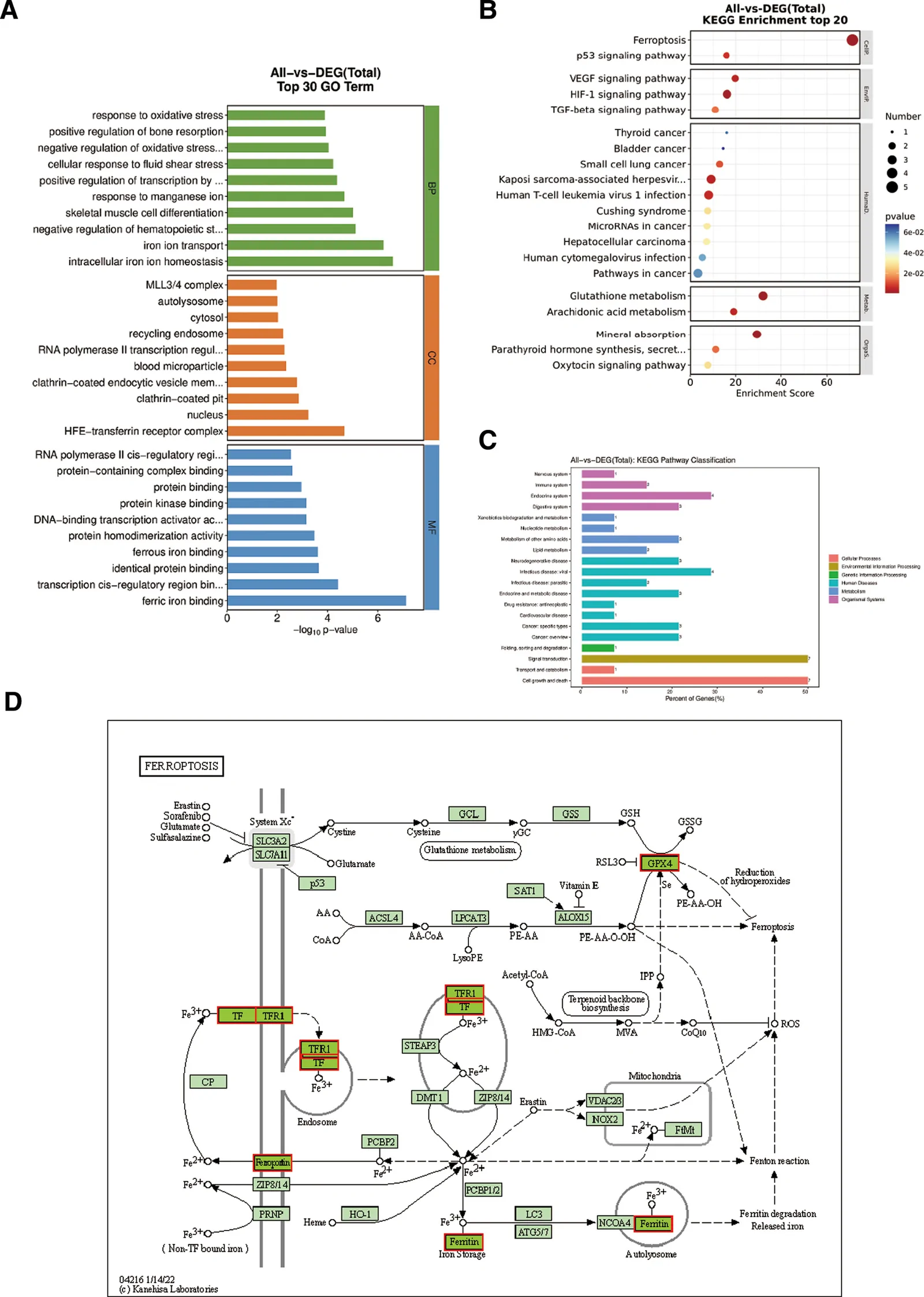
(A) GO enrichment analysis of key genes for iron death, (B) KEGG enrichment analysis of key genes for iron death, (C) KEGG cascade enrichment analysis of key genes for iron death, (D) iron death signaling pathway and key expressed proteins. GO = gene ontology, KEGG = Kyoto encyclopedia of genes and genomes.

The RA Ferroptosis gene collection was subjected to BP analysis, and GO analysis showed that 443 BPs were found for the RA Ferroptosis genes (Table S5, Supplemental Digital Content, https://links.lww.com/MD/Q55). According to the findings, the primary factors influencing blood pressure analysis during RA are intracellular iron-ion homeostasis, iron-ion transport, negative regulation of skeletal muscle cell differentiation, hematopoietic stem cell differentiation, response to manganese ions, positive regulation of transcription by RNA polymerase II, cellular response to fluid shear stress, negative regulation of the intrinsic apoptotic signaling pathway induced by oxidative stress, positive regulation of bone resorption, and reaction to oxidative stress. The primary components of CC analysis during RA are blood particles, circulating endosomes, cytoplasm, autolysosomes, lectin-encapsulated pits, lectin-encapsulated inner vesicle membranes, transferrin receptor complex nuclei, and MLL3/4 complexes. Iron binding, transcriptional cis-regulatory region binding, identical protein binding, ferrous iron binding, protein kinase junction protein binding, and RNA polymerase II cis-regulatory region sequence-specific DNA binding are the primary elements of MF analysis during RA (Fig. 5A). Conversely, KEGG bioprocesses indicate that ferroptosis, glutathione metabolism, mineral uptake, HIF-1 signaling pathway, vascular endothelial growth factor signaling pathway, arachidonic acid metabolism, human T-cell leukemia virus 1 infection, and p53 signaling pathway are all closely linked to the development of RA (Fig. 5B, Table S5, Supplemental Digital Content, https://links.lww.com/MD/Q55). The KEGG signaling pathway hierarchy revealed that RA is strongly linked to the immunological, endocrine, and transport and metabolism systems, as well as cell growth and death (Fig. 5C). Lastly, Ferroptosis is a significant factor in RA, and it further labels critical genes in the Ferroptosis process by querying the KEGG signaling pathway (Fig. 5D).

### 3.5. Immunoassay for RA

**Figure 6.**
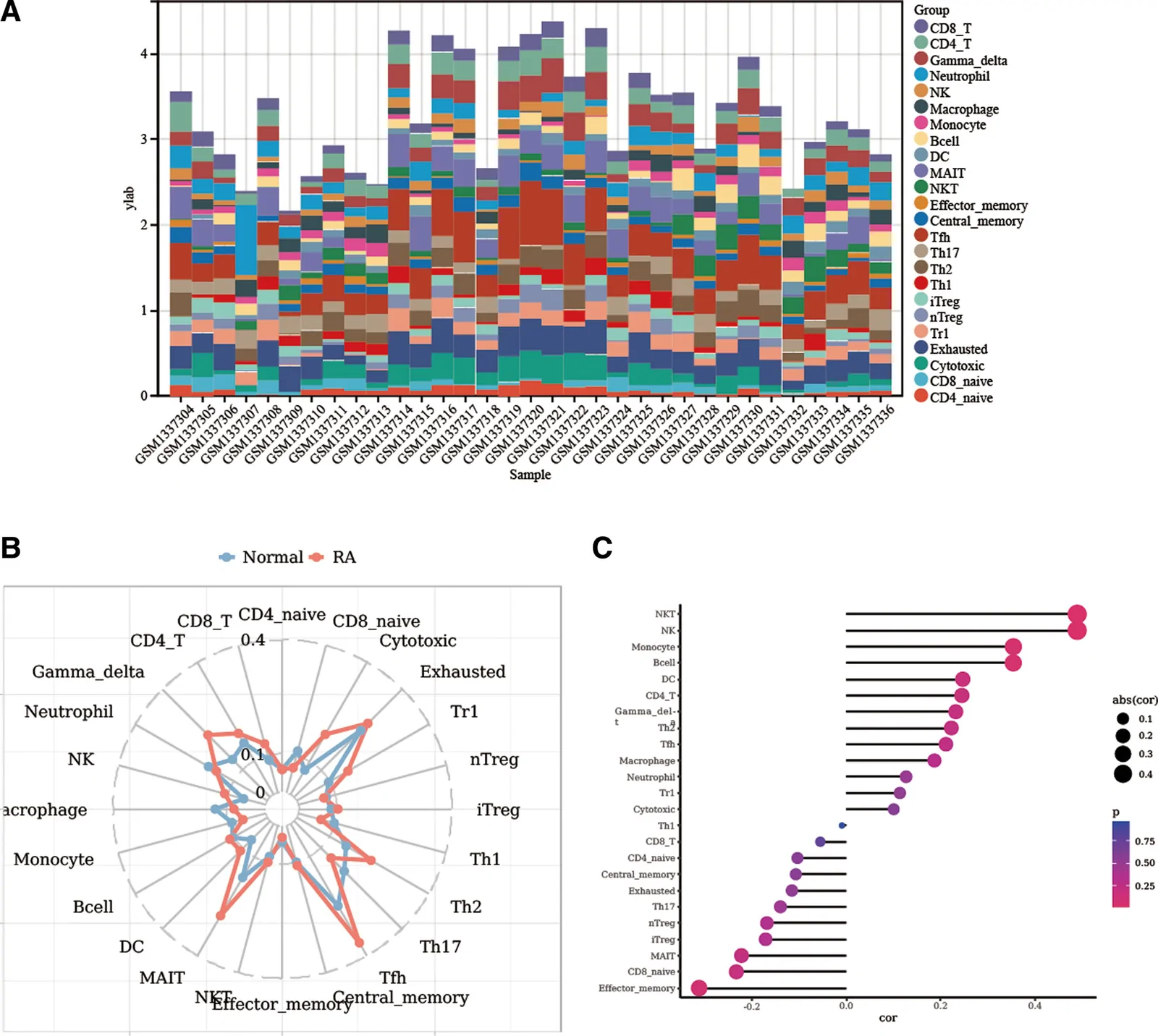
(A) Immuno-infiltration stacking plot, (B) Immunoassay radar trend plot, (C) Immunoassay correlation dot bar plot.

We used the algorithm to determine the immune infiltration scores of the various sample groups, and the findings are displayed in a stacked graph (Fig. 6A, Table S6, Supplemental Digital Content, https://links.lww.com/MD/Q55). The importance of immune cell infiltration in various groups is depicted in Figure 6B, where RA was clearly associated with Gamma_delta, Tfh, and MAIT. NKT, NK, Monocyte, Macrophage, and Neutrophil exhibited positive correlation, while Effectormemory, CD8naïve, MAIT, and iTreg shown negative association, according to the immune cell correlation point bar data. NK, monocytes, macrophages, and neutrophils are the main immune cells involved in the development of inflammation, and it is reasonably certain that they have a positive link with RA (Fig. 6C).

### 3.6. Identification and screening of THSWD components

**Figure 7.**
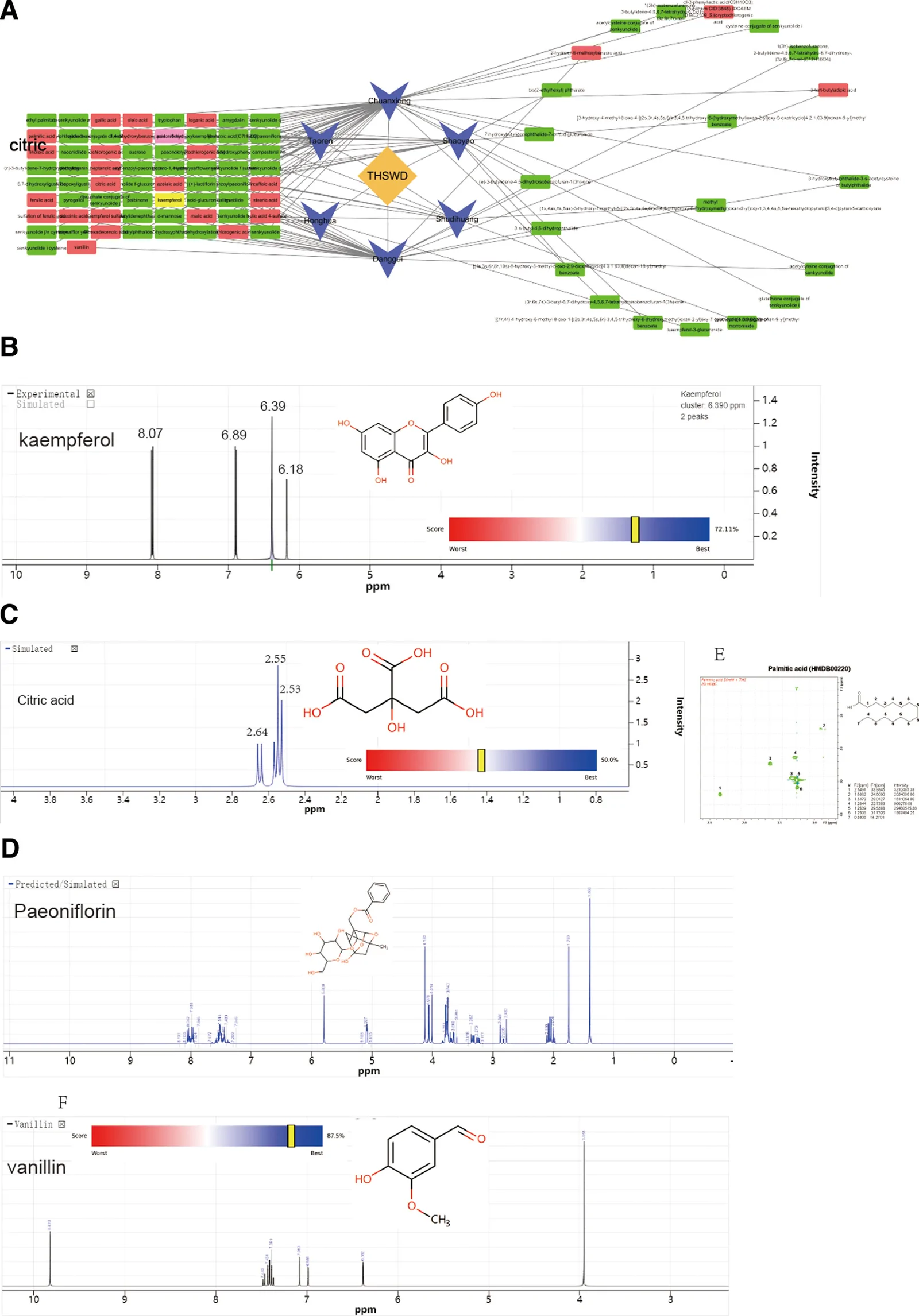
(A) Drug composition network map of THSWD, (B) Mass spectral peaks and chemical formulas and experimental prediction confidence heatmap of kaempferol, (C) Mass spectral peaks and chemical formulas and experimental prediction confidence heatmap of citric acid, (D) mass spectral peaks and chemical formulas of paeoniflorin, (E) palmitic acid mass spectral peaks and chemical formula, mass spectral peak graph and chemical formula of (F) vanillin and experimental prediction confidence heat map. THSWD = Taohong Siwu decoction.

Figure 7A, Table S7, Supplemental Digital Content, https://links.lww.com/MD/Q55 shows that we screened 115 of the main blood-entry components of THSWD using the TCM database. However, by screening and detecting the same ingredients in other medications, we ultimately discovered that the main ingredients for THSWD to produce medicinal effects were palmiticacid, kaempferol, butylphthalide, citric acid, vanillin, and paeoniflorin. We identified these compounds using mass spectroscopy and constructed their chemical formulas in NP-MRD. We ultimately determined that kaempferol was the primary therapeutic molecule among them, with experimental identification confidence scores of 72.11% and 87.5% for kaempferol and vanillin, respectively, when paired with literature screening (Fig. 7B–F).

### 3.7. Molecular mechanisms of THSWD in the treatment of RA

**Figure 8.**
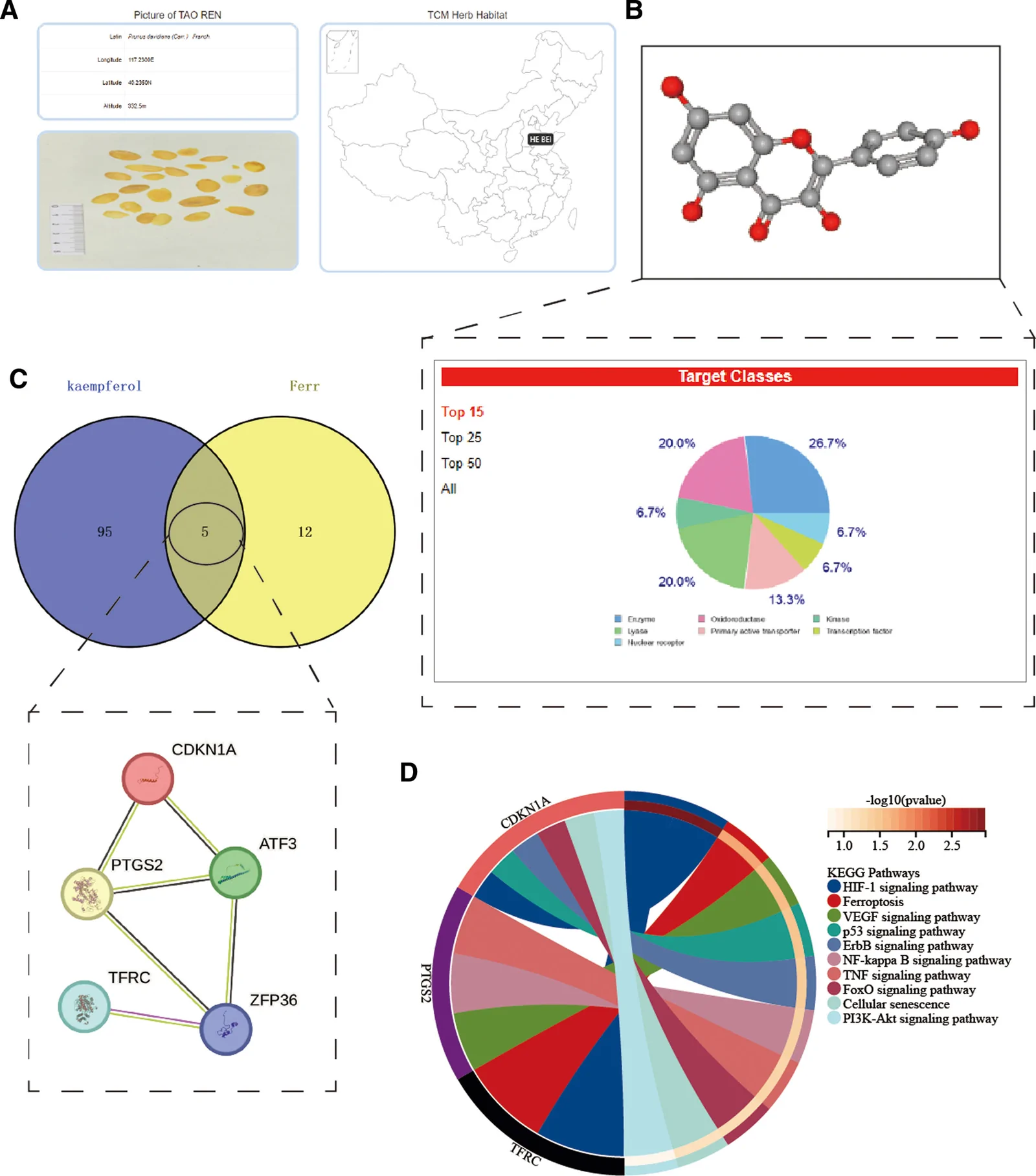
(A) Origin source of the drug TAOREN, the main drug of THSWD, (B) Chemical formula and target action attribution of the main ingredient, kaempferol, (C) Intersection of the target of action of kaempferol with the iron death target of rheumatoid arthritis, and (D) KEGG signaling pathway of kaempferol for rheumatoid arthritis. KEGG = Kyoto encyclopedia of genes and genomes, THSWD = Taohong Siwu decoction.

By searching the TCM database, we discovered that kaempferol was identified in a number of THSWD medications. Additionally, TAOREN, a popular traditional Chinese medicine from Hebei Province, China, was a significant source of kaempferol (Fig. 8A–C). One hundred drug important action targets were ultimately eliminated based on the Swiss target prediction tool’s target prediction of kaempferol (Table S8, Supplemental Digital Content, https://links.lww.com/MD/Q55). Kaempferol target mode of action mostly involves the control of enzymes, oxidative stress, and multiple signal transduction. Five regulatory targets were screened by Kaempferol intersection with RA Ferroptosis targets. The activation target is ATF3, the inhibition target is ZFP36, and the ferroptosis marker genes include PTGS2, TFRC, and CDKN1A.However, the KEGG of these 5 targets once more indicated that the regulation of Ferroptosis, the HIF-1 signaling network, the vascular endothelial growth factor signaling pathway, the p53 signaling system, etc, is strongly linked to the therapy process of RA (Fig. 8D).

### 3.8. In vitro validation results

After CCK-8 screening, we found that kaempferol at 10 μmol/ L concentration showed good value-added effect at both 24 and 48 hours, but the overall trend showed 48 showed unstable cell value-added curve after the intervention. Therefore, we finally selected kaempferol at 10 μmol/ L concentration at 24 hours for subsequent validation.

**Figure 9.**
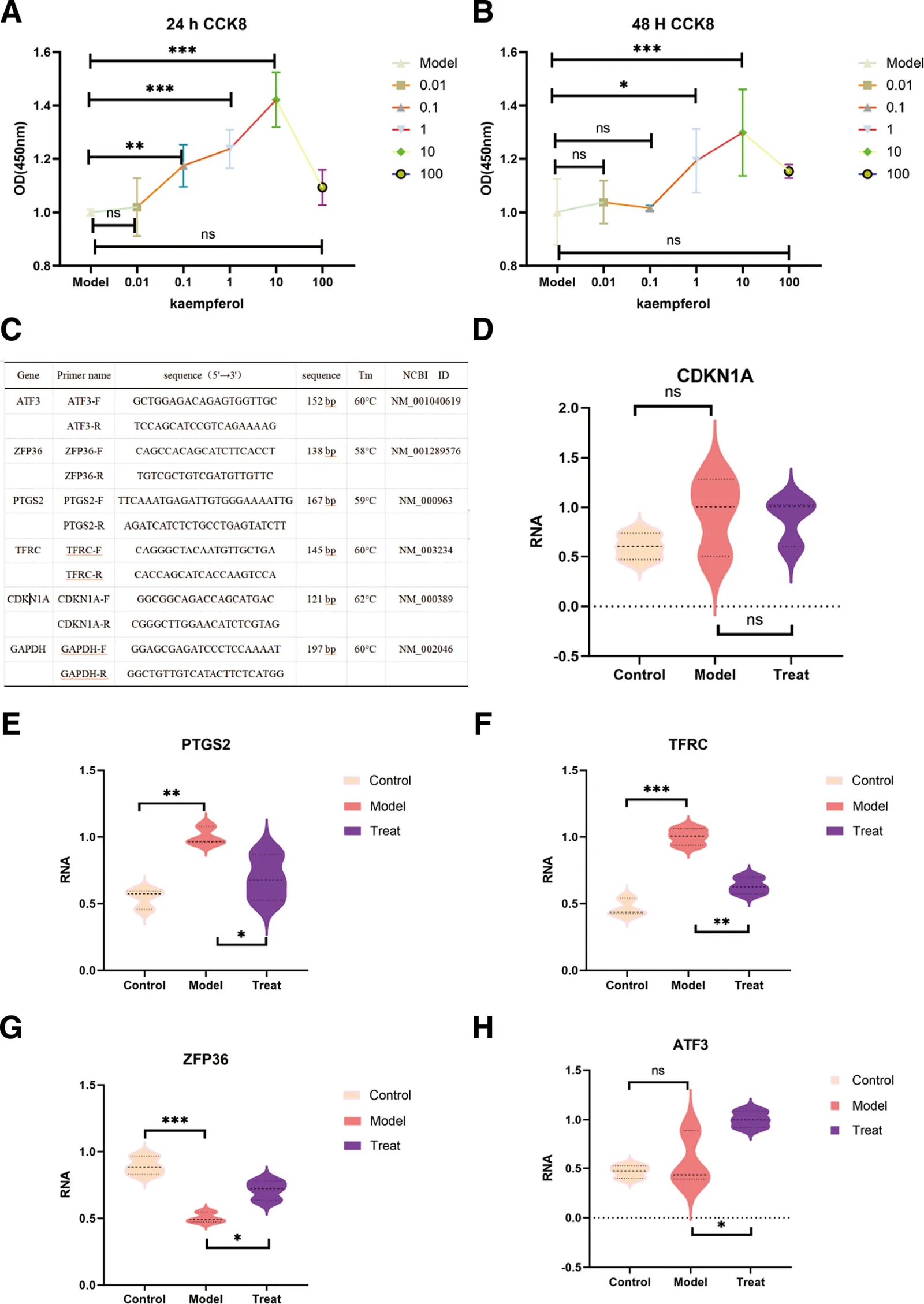
(A) 24-h appreciation curve of sornaferol intervention in human synovial fibroblasts, (B) 48-h appreciation curve of sornaferol intervention in human synovial fibroblasts, (C) ATF3, ZFP36, primer sequence numbers and usage conditions for PTGS2, TFRC and CDKN1A, (D) statistical analysis of RNA expression in the normal, model, and treatment groups of CDKN1A, (E) statistical analysis of RNA expression in the normal, model, and treatment groups of PTGS2, (F) statistical analysis of RNA expression in normal, model, and treatment groups of TFRC, (G) statistical analysis of RNA expression in the normal, model, and treatment groups of ZFP36, (H) statistical analysis of RNA expression in the normal, model, and treatment groups of ATF3.

Kaempferol intervention significantly reversed abnormal gene expression in RA model cells: the pro-inflammatory and Ferroptosis-related genes PTGS2 and TFRC were downregulated in the treatment group (* *P* < .05, ** *P* < .01), while the stress protection factor ATF3 was significantly upregulated (* *P* < .05). Expression of the inflammatory suppressor ZFP36 was partially restored with 10 μmol/L of kaempferol (* *P* < .01). The insignificant statistical change in CDKN1A, which may be related to its presented concentration-dependent regulation, suggests that kaempferol plays a therapeutic effect in concert with Ferroptosis inhibition through dynamic regulation of cell cycle repair. The above changes were significantly associated with the Ferroptosis and HIF-1 pathway predicted by KEGG, confirming that kaempferol improved the RA synoviocyte microenvironment through a multi-target network (Fig. 9A–H).

## 4. Discussion

Ferroptosis is a new type of programmed cell death that is intimately associated with lipid peroxidation, GPX4 activity, iron metabolism, and GSH metabolism. Ferroptosis is essential in the treatment of RA and has a major impact on the prognosis of RA since recent research has demonstrated that it plays a crucial regulatory role in autoimmune and inflammatory illnesses.^[23]^ Research indicates that iron metabolism, system XC-, GPX4 activity, ROS buildup, and other associated signaling pathways can all be used to partially prevent ferroptosis.^[24]^ However, there is still a lack of comprehensive research on the inherent connection between ferroptosis and RA. The Ferroptosis pathway is a possible research avenue for upcoming attempts to find novel treatment targets for RA, given the unparalleled success of Ferroptosis research.^[25]^ Traditional Chinese medicine is based on the idea that various illnesses can be treated with the same medication. The occurrence and progression of important diseases share a common etiology, and various diseases can be treated in tandem. The same concepts of treatment can be used when the pathogenesis is the same. Osteoarthritis is typically categorized as “bone obstruction,” “obstruction syndrome,” “muscle obstruction,” etc in traditional Chinese medicine. According to traditional Chinese medicine, both RA and “bone obstruction” fall within the “obstruction syndrome” category. To clarify the possible mechanisms of THSWD’s “treating different diseases with the same treatment” for RA, this study employs bioinformatics and network pharmacology to examine the protein genes, active chemical components, and associated BPs of RA.^[10]^ The Ferroptosis marker gene PTGS2 SLC40A1, TF, TFRC, FTH1, GPX4, HSPB1, NFE2L2 has been found based on the identification of differentially expressed genes, module genes, and genes related to the Ferroptosis process. Among these, the sample tissues exhibit high expression of TFRC, FTH1, GPX4, HSPB1, and NFE2L2. Furthermore, we discovered 3 ferroptosis activators, specifically ATF3, EGR1, and KDM6B. Ferroptosis is inhibited by ZFP36 NR4A1 CDKN1A ABRD4 RRM2. The tissues in the sample express ZFP36, CDKN1A, and NR4A1. The disease process of RA is linked to intracellular iron-ion balance, iron-ion transport, skeletal muscle cell differentiation, manganese ion response, fluid shear stress cell response, negative regulation of intrinsic apoptosis signaling pathways induced by oxidative stress, and response to oxidative stress, according to additional biological data analysis. Iron binding, transcriptional cis-regulatory region binding, homologous protein binding, ferrous binding, protein kinase binding, and RNA polymerase II cis-regulatory region sequence-specific DNA binding are the primary MF analysis processes during the RA process. According to the KEGG BP, RA is intimately associated with Ferroptosis, the p53 signaling system, the vascular endothelial growth factor signaling pathway, the HIF-1 signaling pathway, and others. Lastly, we discovered via network pharmacology that kaempferol, the main ingredient in THSWD, improves RA by controlling ferroptosis via TFRC, ZFP36, PTGS, CDKN1A, and ATF3.^[26]^

Excessive increase in iron concentration in the body can lead to membrane lipid peroxidation and cell death.^[25]^ Studies have demonstrated that Fe3 + is liberated into the cytoplasm and transformed into Fe2+, which takes part in the Fenton reaction, generating an excess of ROS and resulting in ferroptosis. The majority of research has demonstrated that altering the cells’ unstable iron level can change how susceptible they are to ferroptosis. Iron regulatory proteins play a major role in maintaining the equilibrium of cellular iron metabolism, and transferrin (TF) and its receptors cooperate to deliver iron into cells in order to induce ferroptosis. By boosting iron uptake, overexpression of TF and TFR1 increases ferroptosis sensitivity, whereas TFR1 silencing can prevent ferroptosis.^[27,28]^ Prior research has demonstrated that by suppressing TRF1 expression, heat shock protein B1 (HSPB1) dramatically prevents ferroptosis by lowering intracellular iron content. According to a study, ubiquitin-specific protease 7 can promote ferroptosis in rats’ hearts during ischemia-reperfusion therapy by increasing iron absorption through the activation of the p53/TFR1 pathway.^[29]^ Consequently, iron overload and ferroptosis can result from increased iron intake caused by TFR1 overexpression. Additionally, we discovered that kaempferol contributes to ferroptosis via TFRC. Based on this, we anticipate that kaempferol can inhibit TFRC to regulate iron intake, thus ameliorating ferroptosis in RA.

ATF3 imbalance has been linked in studies to atherosclerosis, cancer, infection, and inflammation, among other illnesses. In an endotoxin-treated mouse model, ATF3 interacts with NF-κ B to produce immunomodulatory effects by blocking pro-inflammatory cytokines such IL-6, IL-12b, and toll-like receptor 4.^[30]^ Studies have demonstrated that in mouse bone marrow macrophages, ATF3 deficiency can reverse the production and proliferation of osteoclasts, mediating the inhibitory effect of TNF-α on osteoblast differentiation.^[30,31]^ According to the research mentioned above, ATF3 is an adaptive response gene that can have either boosting or inhibiting effects. Furthermore, research has shown that ATF3 protects the myocardium and reduces ferroptosis brought on by cardiac I/R. This may be because ATF3 directly binds to the transcription start site of the ferroptosis inhibitor gene FANCD2 and promotes its transcription.^[32]^ ATF3 was discovered to encourage ferroptosis brought on by erastin. ATF3 promotes erastin-induced lipid peroxidation by inhibiting system Xc- and depleting intracellular GSH.ATF3 accomplishes this function by attaching itself to the promoter of SLC7A11 and p53-independently suppressing SLC7A11 expression. activity by attaching itself to the SLC7A11 promoter and p53-independently suppressing SLC7A11 expression.^[32]^ In conclusion, ATF3 is an activating factor leading to RA, and kaempferol may act by inhibiting ATF3.

According to a study, kaempferol protects H9C2 cardiomyocytes by triggering the Nrf2/GPX4 signaling pathway and preventing H/R injury-induced ferroptosis in these cells.^[33,34]^ It is evident that by interacting with important genes like GPX4, kaempferol can control ferroptosis. However, more research is required to determine how kaempferol controls ferrotosis in RA. In vivo experiments demonstrated that THSWD effectively attenuated brain injury and restored neural function in MCAO/ R rats. THSWD Significant enhanced CD31, Ang1, PDGFB and PDGFR- β expression levels, possibly related to improved glucose, pyruvate and ATP levels, along with decreased lactate and lactate/ pyruvate ratio. The in vitro findings suggest that THSWD may accelerate mature angiogenesis by promoting the expression of mature angiogenic factors (VE GF A, Ang1, and PPDGFB) by activating glycolysis, increasing glucose uptake, and increasing lactate, pyruvate, and ATP content. THSWD Can relieve CIRI by activating the glycolytic pathway to promote mature angiogenesis. Targeting glycolysis-mediated mature angiogenesis together with THSWD, the treatment holds hope for IS therapy. Therefore, this effect may be instructive in treating RA.^[35,36]^ The pathogenesis of orthopedic diseases is closely related to blood stasis and is usually caused by primary and secondary blood channel damage. This interruption can lead to “blood outlet meridians” or air stagnation, which leads to blood stasis syndrome. THSWD is a well-known classic TCM formula, which is widely used to promote blood circulation and relieve blood stasis. Clinical studies have shown that it has significant therapeutic effects in various orthopedic diseases, especially its anti-inflammatory and analgesic properties, and the efficacy of preventing postoperative deep vein thrombosis. Despite these findings, research on THSWD remains fragmented and its interdisciplinary impact is limited. In the current study, it was found that THSWD may improve RA by regulating chondrocyte iron death, which will provide new insights into the application of THSWD in orthopedic diseases.^[36]^ There are several shortcomings in this study. The first is that the number of Ferroptosis-activating or inhibiting genes obtained from this bioinformatics is low, and a large number of target screening is still needed compared to numerous other studies. The second is that the screening of drug components based on compounding is not accurate enough and lacks real-world mass spectrometry data validation and ex vivo and in vivo studies. Thirdly, there are few reports on the mechanism of Ferroptosis modulation by kaempferol, especially in RA.

## 5. Conclusion

We determined the main targets of ferroptosis in RA using bioinformatic analysis; they were ZFP36, NR4A1, CDKN1A, ABRD4, and RRM2 as inhibitors and ATF3, EGR1, and KDM6B as activation targets. We discovered from the findings of the enrichment study that the pathogenic process of RA is directly linked to the transport of iron ions, ferroptosis, the HIF signaling pathway, etc. Lastly, using network pharmacology, we discovered that kaempferol, the main ingredient in THSWD, enhanced the Ferroptosis process of RA by targeting several different targets. Therefore, the present study reveals the molecular mechanism of Ferroptosis in the treatment of RA with THSWD.THSWD can provide a promising direction for the future treatment of RA, but it needs to be validated by sufficient relevant experimental studies.

## Author contributions

**Conceptualization:** Ying Shi.

**Data curation:** Hang Du.

**Funding acquisition:** Ying Shi.

**Methodology:** Yue Ren.

**Software:** Qu Zhang.

**Writing – original draft:** Ping Xia.

## Supplementary Material
